# Trimming flow, plasticity, and mechanical properties by cubic silsesquioxane chemistry

**DOI:** 10.1038/s41598-023-40784-4

**Published:** 2023-08-29

**Authors:** Bogna Sztorch, Dariusz Brząkalski, Julia Głowacka, Daria Pakuła, Miłosz Frydrych, Robert E. Przekop

**Affiliations:** 1 Centre for Advanced Technologies, Adam Mickiewicz University in Poznań, 10 Uniwersytetu Poznańskiego, 61−614 Poznan, Poland; 2 Faculty of Chemistry, Adam Mickiewicz University in Poznań, 8 Uniwersytetu Poznańskiego, 61−614 Poznan, Poland

**Keywords:** Engineering, Materials science

## Abstract

In this work, the possibility of managing the rheological and mechanical parameters of composites based on PLA with the use of cubic structures of organofunctional spherosilicates was verified. To accurately observe the effect of various organosilicon modifier substitutions on changes in composites’ properties, we synthesized and used monofunctional octasubstituted derivatives as reference systems. The OSS/PLA systems were tested with concentrations of 0.1–2.5% (w/w) using extrusion to obtain a filament with a diameter of 1.75 mm. The printed samples underwent comprehensive tests including microscopic (SEM–EDS, optical microscope), rheological, thermal (TG, DSC, HDT), mechanical (impact and strength) as well as water contact angle tests. The work is interdisciplinary in nature and combines elements of organosilicon synthesis, materials engineering, and materials processing and characterization technology.

## Introduction

The dynamic development of additive techniques occurred in the second half of the 80s. Then, after the expiry of the original patent on FDM (2009) and SLS technology (2014), there was a wave of new companies offering equipment and materials dedicated to 3D printing. It was also associated with a decrease in 3D printer prices and the related increase in the number of scientific publications and new patents in this area. Currently, 3D printing is one of the most dynamically developing industries. Both competitive pressures and increasingly sophisticated business models mean that companies need to shorten development cycles and move in the right direction with speed and flexibility. 3D printing in many industries is revolutionizing production thanks to more materials available and a greater ability to supply parts that match their mechanical properties. Currently, incremental techniques are increasingly replacing other mature processing methods, while the demand for 3D printing results is constantly growing^1^. Additive technologies are recognized as one of the key elements of the new generation of the 4.0 Industry. SmartTech Analysis is currently a leader among analytical companies in the field of additive technologies. It mainly focuses on forecasting the economic impact of 3D printing, which in 2020 was 11.7 billion dollars. In subsequent years, it is forecasted at USD 24 billion in 2024 and USD 55 billion in 2030, respectively. The development of additive technologies depends on the industry's demand, mainly in terms of automation and optimization of processes. The most progress has been made in the sector of designing and manufacturing new materials. The latest SmartTech Analysis report shows that the largest revenues until 2019 were generated by FDM technology^2^. It is expected that the greatest revenues among the known and applied incremental methods are generated by the FDM technology, mainly due to its low complexity and commercial access to raw materials (filaments)^2^. Currently, FDM printers are available not only for large corporations but also for micro-enterprises and individual customers. Device prices come in a very wide range, on the market There is also a wide range of products (in terms of color and flexibility modification) and a database of millions of ready-made designs, which greatly simplifies the process.

**Figure. 1:**
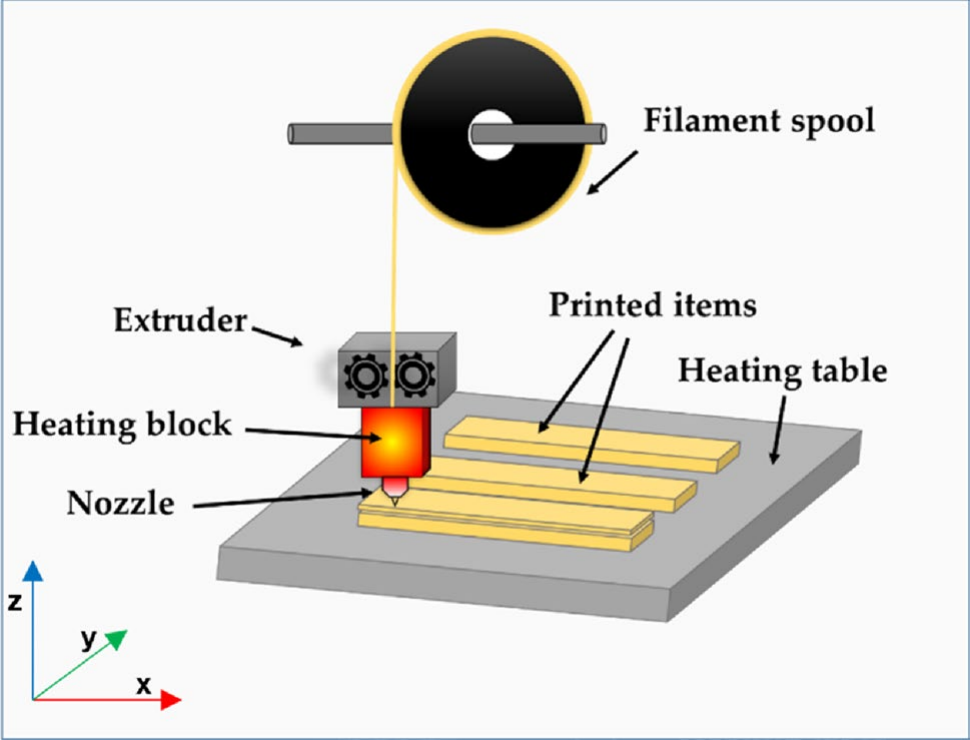
Scheme of an FDM 3-D printer.

The FDM technique is a kind of micro-extrusion, it consists in feeding molten thermoplastic material from the printer's nozzle and then applying it layer by layer on a movable table^3^. The general scheme of a typical FDM 3-D printer was presented in Fig. 1. The most commonly used polymers in the FDM technique include polylactic acid (PLA)^4–7^ poly (ethylene terephthalate) glycol modified (PET-G)^8,9^, amorphous acrylonitrile butadiene styrene (ABS)^10–12^ and polyamide-12 (PA-12)^13,14^.

The importance of 3D printing to other technologies is very significant due to the many benefits that the technique brings, e.g. short time from design to prototype execution^15^, shortening the supply chain, and enabling spare parts manufacturing in production plants^16^, and the ability to produce even very complex structures (without the need to create many expensive forms)^17^.

The greatest limitation of the FDM technique is the lack of highly specialized materials that would allow the elimination of such limitations as adhesion to the layer and the printer bed, or improvement of performance properties without compromising others. Printing issues often arise from imperfections in print tightness and hydrophobic and/or mechanical and thermal properties. So far, the greatest emphasis has been placed on the development of new devices with higher printing speeds, larger working area, etc. There are several cases in the literature in which thermoplastic polymers used in the FDM technique have been modified to improve the quality of printing or the properties of model objects. There are known works where the authors created a SiC/C/PLA composite characterized by good electrical conductivity and shape memory^18^. The addition of glass fibers and a plasticizer/compatibilizer as a modifier of the mechanical properties of composites based on ABS (the addition of the fibers themselves causes too rigid a structure and contributes to the difficulty of the printing process—clogging and abrasion of the nozzle)^19^ or use to improve the mechanical properties at the boundary of the matrix and glass fibers, carbon or Kevlar^20^. To a large extent, the improvement of the properties of materials popularly used in 3D printing is based on physical modification with the use of reinforcements in the form of synthetic or natural fibers^21^. Metal nanoparticles are also often used as reinforcing additives^22,23^. The addition of graphite improved the rheological and tribological properties^24^. In our previous work, we also examined the effect of the addition of MoS_2_^25^ which showed a change in tribological properties, and TiO_2_, which improved the mechanical properties of the obtained composites^26^. So far, however, there are no solutions that would enable the improvement of several parameters at the same time or would increase the desired parameters without drastically lowering others.

One of the most attractive and increasingly used groups of modifiers of various classes of polymers is polyhedral oligomeric silsesquioxanes with the general formula [RSiO_1,5_]_n_^27^. Thanks to their structure, i.e. an inorganic core made of Si–O–Si connections and organic substituents at the silicon atom, they have organic–inorganic hybrid properties^28,29^. Silsesquioxanes can be used as additives in the production of thermoplastic or thermosetting polymers as well as composite materials. They are used in optoelectronics, microelectronics, pharmacy (drug carriers), medicine, and dentistry^30–32^. Octasubstituted silsesquioxanes are an especially interesting group. They are obtained by a simple hydrolytic condensation reaction of such compounds as trichloalkoxysilanes or trialkoxysilanes^29,33,34^. Silsesquioxanes, due to their compact molecular structure and nanometric dimensions, are characterized by good dispersion properties. A special subgroup of cage silsesquioxanes, sometimes treated as a separate class of compounds, are spherosilicates, which are characterized by an additional D-type siloxane linker, separating the R functional group from the siloxane core, composed of Q-type units. These structures are characterized by high activity in catalytic reactions, among which the hydrosilylation of olefins is of the greatest importance, due to the large variety and availability of substrates (also in economic terms), the quantitative nature of the reaction, and the ease of the synthetic procedure. These characteristics determine the growing interest in compounds of the spherosilicate type as functional additives in materials engineering^32,35,36^ or building blocks in polymer chemistry^37^.

The purpose of this work was to assess the properties of spherosilicate/PLA composite materials obtained by polymer compounding methods and dedicated to the FDM 3D printing technique. The material was modified with a series of mono- and bifunctional octaspherosilicate (OSS) additives obtained by a hydrosilylation reaction. Modifiers were tested and characterized by means of spectroscopy and spectrometry (FT-IR, MALDI-TOF–MS, NMR). The conducted tests of composite materials revealed that the additives strongly affect the PLA melt rheology, polymer fusion during printing, material delamination, fracture mode under mechanical load, and therefore mechanical strength of the printed samples. Additionally, the thermal and surface properties of the obtained materials have been affected by the OSS compounds.

## Experimental

### Materials

Polylactide (PLA) type Ingeo 2003D was purchased from NatureWorks (Minneapolis, MN, USA). The chemicals were purchased from the following sources: Tetraethoxysilane (TEOS), chlorodimethylsilane, tetramethylammonium hydroxide (TMAH) 25 wt% methanol solution from ABCR, vinyltrimethoxysilane, octadec-1-ene from Linegal Chemicals (Warsaw, Poland) chloroform-d, 2 wt% Karstedt’s catalyst xylene solution from Aldrich (Poznan, Poland), P2O5, toluene, from Avantor Performance Materials Poland S.A. (Gliwice, Poland). Toluene was degassed and dried by distilling it from P2O5 under an argon atmosphere.

## Methods

### Analyses

^1^H, ^13^C, and ^29^Si Nuclear Magnetic Resonance (NMR) spectra were recorded at 25°C on Bruker Ascend 400 and Ultra Shield 300 spectrometers using CDCl_3_ as a solvent. Chemical shifts are reported in ppm concerning the residual solvent (CHCl_3_) peaks for ^1^H and ^13^C.

MALDI-TOF mass spectra were recorded on an UltrafleXtreme mass spectrometer (Bruker Daltonics), equipped with a SmartBeam II laser (355 nm) in the 500–4000 m/z range. 2,5-Dihydroxybenzoic acid (DHB, Bruker Daltonics, Bremen, Germany) served as a matrix. Mass spectra were measured in reflection mode. The data were analyzed using the software provided with the Ultraflex instrument—FlexAnalysis (version 3.4).

Fourier Transform-Infrared (FT-IR) spectra were recorded on a Nicolet iS 50 Fourier transform spectrophotometer (Thermo Fisher Scientific) equipped with a diamond ATR unit with a resolution of 0.09 cm^−1^.

Water contact angle analyses (WCA) were performed by the sessile drop technique at room temperature and atmospheric pressure, with a Krüss DSA100 goniometer Three independent measurements were performed for each sample, each with a 5 µl water drop, and the obtained results were averaged to reduce the impact of surface non-uniformity.

Thermogravimetry (TG) was performed using a NETZSCH 209 F1 Libra gravimetric analyzer. Samples of 5 ± 0.2 mg were cut from each granulate and placed in Al_2_O_3_ crucibles. Measurements were conducted under nitrogen (flow of 20 mL/min) in the range of 30 ÷ 800 °C and a 10 °C/min heating rate, Sensitivity: 0.1 µg.

Differential scanning calorimetry (DSC) was performed using a NETZSCH 204 F1 Phoenix calorimeter Samples of 6 ± 0.2 mg were cut from each granulate and placed in an aluminum crucible with a punctured lid. The measurements were performed under nitrogen in the temperature range of − 20 ÷ 290°C and at a 10 °C/min heating rate., Sensitivity: 0.1 µg.

The effect of the modifier addition on the mass flow rate (MFR) was also determined. The measurements were made using an Instron plastometer, model Ceast MF20 according to the applicable standard ISO 1133. The measurement temperature was 190 ± 0.5 °C, while the piston loading was 2.16 kg.

For flexural and tensile strength tests, the obtained materials were printed into type 1B dumbbell specimens by EN ISO 527:2012 and EN ISO 178:2006. Tests of the obtained specimens were performed on a universal testing machine INSTRON 5969 with a maximum load force of 50 kN. The traverse speed for tensile and for flexural measurements was set at 2 mm/min. Charpy impact test (with no notch) was performed on an Instron Ceast 9050 impact machine according to ISO 179−1. For all the series, 6 measurements were performed for each material.

Scanning Electron Microscopy with Energy Dispersive Spectroscopy (SEM/EDS) analyses were recorded on a Quanta FEG 250 (FEI) instrument; SEM at 5 kV and EDS at 30 kV.

Surface structure and breakthroughs were analyzed under Digital Light Microscope Keyence VHX 7000 with 100- × 1000 VH-Z100T lens. All of the pictures were recorded with a VHX 7020 camera.

### The procedure for synthesis of Octadecyl-trimethoxysilyl octaspherosilicate derivatives (OSS-8OD, OSS-5OD-3TMOS, OSS-4OD-4TMOS, OSS-2OD-6TMOS, OSS-8TMOS

The hydrosilylation reaction was performed according to a previous report^38^. In a typical procedure, a 500 mL three-neck, round-bottom flask was charged with 25.00 g of Octahydrospherosilicate (24.56 mmol), 250 ml of toluene, and olefins, that is, vinyltrimethoxysilane (VTMOS) and octadec-1-ene (OD) in a molar ratio according to the stoichiometry of the product to be obtained (from 8:0 to 0:8 of VTMOS:OD, the amount of the olefin in total adding up to 197 mmol). A magnetic stirring bar was added. A thermometer and condenser equipped with an argon inlet and oil bubbler were attached, the flask was placed in a heating mantle and the system was purged with argon. The reaction mixture was set at 110°C and before reaching boiling, 25 µl of Karstedt’s catalyst solution was added, which resulted in a quick increase of temperature and the system starting to reflux. The reaction mixture was kept at reflux and samples were taken for FT-IR control until full Si–H group consumption was observed. After reaction completion (usually ~ 24h) solvent was evaporated under a vacuum to dry to obtain the final product. OSS-8TMOS and OSS-2OD-6TMOS were obtained as clear liquids of relatively low viscosity, OSS-4TMOS-4OD, and OSS-3TMOS-5OD as soft, white waxes, and OSS-8OD as hard, white wax.

### OSS-8OD 1,3,5,7,9,11,13,15-octa(dimethyl(octadecyl)siloxy)-pentacyclo[9.5.1.1^3,9^.1^5,15^.1^7,13^]octasiloxane)

^**1**^**H NMR** (400 MHz, CDCl_3_): d (ppm) = 1.31–1.26 (m, 256H, –CH_2_- groups), 0.88 (t, 24H, –CH_2_CH_3_), 0.59 (t, 16H, SiCH_2_-), 0.12 (s, 48H, SiMe_2_);

^**13**^**C NMR** (101 MHz, CDCl_3_): d (ppm) = 33.70, 32.11, 32.10, 29.99, 29.96, 29.94, 29.93, 29.92, 29.91, 29.87, 29.86, 29.65, 29.55, 23.18, 22.87, 18.08, 17.89, 14.28 (–CH_2_CH_3_), − 0.17 (SiMe_2_);

^**29**^**Si NMR** (79,5 MHz, CDCl_3_): d (ppm) = 12.58 (OSiMe_2_), -108.88 (core)*.*

### OSS-5OD-3TMOS 1,3,5,7,9,11,13,15-)-tetra(dimethyl(octadecyl)siloxy)-tetra((trimethoxysilyl)ethyldimethylsiloxypenta- cyclo[9.5.1.1^3,9^.1^5,15^.1^7,13^]octasiloxane

^**1**^**H NMR** (400 MHz, CDCl_3_): d (ppm) = 3.56 (OMe), 1.30–1.25 (m, –CH_2_- groups), 0.88 (t, –CH_2_CH_3_), 0.61–0.56 (m, SiCH_2_-) 0.13 (s, (trimethoxysilyl)ethyl SiMe_2_), 0,12 (s, octadecyl SiMe_2_) ;

^**13**^**C NMR** (101 MHz, CDCl_3_): d (ppm) = 51.00, 50.75, 50.69, 50.50 (OMe), 33.68, 32.10, 29.95, 29.92, 29.90, 29.84, 29.64, 29.54, 23.16, 22.86, 17.88, 14.27 (octadecyl), 8.64 (Si–CH_2_CH_2_-Si), 7.41, 5.27 (SiCH(CH_3_)Si), 0.40 (Si–CH_2_CH_2_-Si), − 0.18, − 0.21, (octadecyl SiMe_2_), − 0.95, − 0.98, − 1.00 ((trimethoxysilyl)ethyl SiMe_2_);

^**29**^**Si NMR** (79,5 MHz, CDCl_3_): d (ppm) = 13.21–12.59 (OSiMe_2_), − 41.55 (Si(OMe)_3_), − 108.88-(−108.98) (core).

### OSS-4OD-4TMOS* 1,3,5,7,9,11,13,15-)-penta(dimethyl(octadecyl)siloxy)-tri((trimethoxysilyl)ethyldimethylsiloxypenta-cyclo[9.5.1.1*^***3,9***^***.1***^***5,15***^***.1***^***7,13***^***]octasiloxane***

^**1**^**H NMR** (400 MHz, CDCl_3_): d (ppm) = 3.55 (OMe), 1.30–1.25 (m, –CH_2_- groups), 0.88 (t, –CH_2_CH_3_), 0.61–0.56 (m, SiCH_2_-) 0.13 (s, (trimethoxysilyl)ethyl SiMe_2_), 0,12 (s, octadecyl SiMe_2_) ;

^**13**^**C NMR** (101 MHz, CDCl_3_): d (ppm) = 50.99, 50.73, 50.69, 50.51 (OMe), 33.68, 32.11, 29.96, 29.94, 29.91, 29.86, 29.66, 29.55, 23.16, 22.87, 17.86, 14.28 (octadecyl), 8.63 (Si–CH_2_CH_2_-Si), 7.42, 5.29 (SiCH(CH_3_)Si), 0.41 (Si–CH_2_CH_2_-Si), − 0.18, − 0.21, (octadecyl SiMe_2_), − 0.95, − 0.97, − 1.02 ((trimethoxysilyl)ethyl SiMe_2_);

^**29**^**Si NMR** (79,5 MHz, CDCl_3_): d (ppm) = 13.22–12.59 (OSiMe_2_), -41.55 (Si(OMe)_3_), -108.88-(-108.98) (core).

### OSS-2OD-6TMOS *1,3,5,7,9,11,13,15-)-di(dimethyl(octadecyl)siloxy)-hexa((trimethoxysilyl)ethyldimethylsiloxypenta-****cyclo[9.5.1.1***^***3,9***^***.1***^***5,15***^***.1***^***7,13***^***]octasiloxane***

^**1**^**H NMR** (400 MHz, CDCl_3_): d (ppm) = 3.56 (OMe), 1.30–1.25 (m, –CH_2_- groups), 0.88 (t, -CH_2_CH_3_), 0.61–0.56 (m, SiCH_2_-) 0.13 (s, (trimethoxysilyl)ethyl SiMe_2_), 0,12 (s, octadecyl SiMe_2_) ;

^**13**^**C NMR** (101 MHz, CDCl_3_): d (ppm) = 50.98, 50.75, 50.68, 50.50 (OMe), 33.67, 32.10, 29.90, 29.84, 29.64, 29.54, 23.16, 22.86, 17.86, 14.26 (octadecyl), 8.94, 8.63(Si–CH_2_CH_2_-Si), 7.41, 5.27 (SiCH(CH_3_)Si), 0.41 (Si–CH_2_CH_2_-Si), − 0.18, − 0.21, − 0.23 (octadecyl SiMe_2_), − 0.95, − 0.98, − 1.00, − 1.06 ((trimethoxysilyl)ethyl SiMe_2_);

^**29**^**Si NMR** (79,5 MHz, CDCl_3_): d (ppm) = 13.20–12.58 (OSiMe_2_), − 41.57 (Si(OMe)_3_), − 108.95–(− 108.98) (core).

### OSS-8TMOS* 1,3,5,7,9,11,13,15-octa((trimethoxysilyl)ethyldimethylsiloxy)-pentacyclo[9.5.1.1*^3,9^.1^5,15^.1^7,13^] octasiloxane, mixture of alpha and beta isomers

^**1**^**H NMR** (400 MHz, CDCl_3_): d (ppm) = 3.55 (s, 72H, OMe), 1.11 (d, alpha product –CH_3_) 0.59 (s, SiCH_2_CH_2_Si), 0.13 (s, 48H, SiMe_2_);

^**13**^**C NMR** (101 MHz, CDCl_3_): d (ppm) = 50.67 (OMe), 8.58 (Si–CH_2_CH_2_-Si), 7.36, 5.25 (SiCH(CH_3_)Si), 0.41 (Si–CH_2_CH_2_-Si), − 1.05 (SiMe_2_);

^**29**^**Si NMR** (79,5 MHz, CDCl_3_): d (ppm) = 13.23 (OSiMe_2_), − 41.66, − 42.78 (OSi(OMe)_3_), − 108.95 (cage).

### Fabrication of filaments. Preparation of granulates

The polymer and the chosen additive were homogenized using a laboratory two-roll mill ZAMAK MERCATOR WG 150/280. A portion of 500 g PLA Ingeo™ 2003 D was heated on the rolls until molten, and then the additive was slowly added in portions, until the final concentration of 5.0 wt.% (with respect to the whole system mass). The mixing was performed at the roll temperature of 210 °C for 15 min., getting to full homogeneity of the composition. The resulting polymer system was then granulated using a SHINI SG-1417-CE grinding mill and dried at 55 °C/24 h. The granulates were diluted with neat PLA up to the final additive loading of 0.10, 0.25, 0.50, 1.0, 1.5, 2.5 wt.% upon screw extrusion with subsequent cold granulation on the single-screw extrusion setup FILABOT EX6, and dried for 24 h at 40 °C.

### Extrusion of filaments

The granulates obtained as above were used for the extrusion of filaments of 1.75 mm diameter by a single-screw extrusion setup FILABOT EX6. Extrusion temperatures were sequentially from the nozzle to the feed zone respectively: 170 °C, 195 °C, 190 °C, and 60 °C.

### 3D printing (FDM)

**Figure 2:**
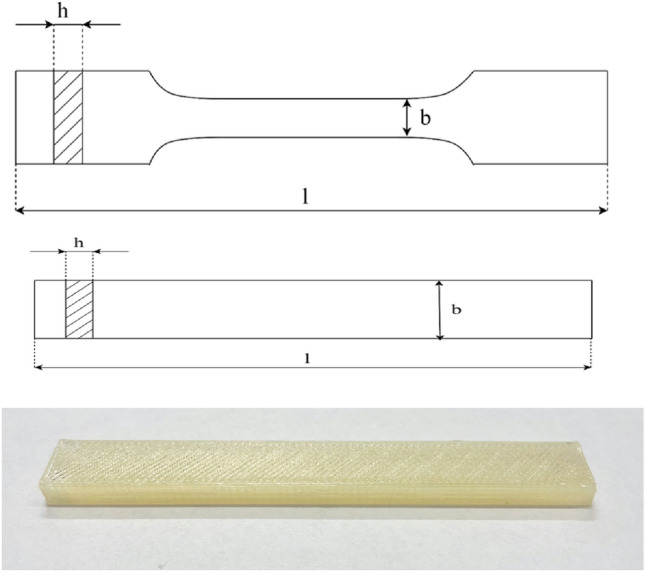
Dimensions of samples used for the mechanical tests: dumbbells—total length l = 80 ± 2 mm, thickness h = 4.0 ± 0.2 mm, the width of the measuring part b = 10.0 ± 0.2 mm, for mechanical tests and microscopic observation: bars—total length l = 80 ± 2 mm, thickness h = 4.0 ± 0.2 mm, the width of the measuring part b = 10.0 ± 0.2 mm,

Using a 3D printer Prusa i3 MK3S + two types of samples were printed by FDM: dumbbells and bars, according to PN-EN- ISO 527−2. The parameters of printing are given in Table 1, Fig. 2.

**Table 1:** Process parameters for sample printing.

Layer height	0.18 mm
Top layer height	0.27 mm
Shells	2
Top and bottom layers number	3
Bottom layers number	3
Infill density	100%
Fill angle	45°
Infill pattern	Rectilinear grid
Printing speed	60 mm/s
Idle speed	80 mm/s
Extruder temp	220 °C
Table temp	60 °C

## Results and discussion

### Characterization of synthesis products

**Figure 3:**
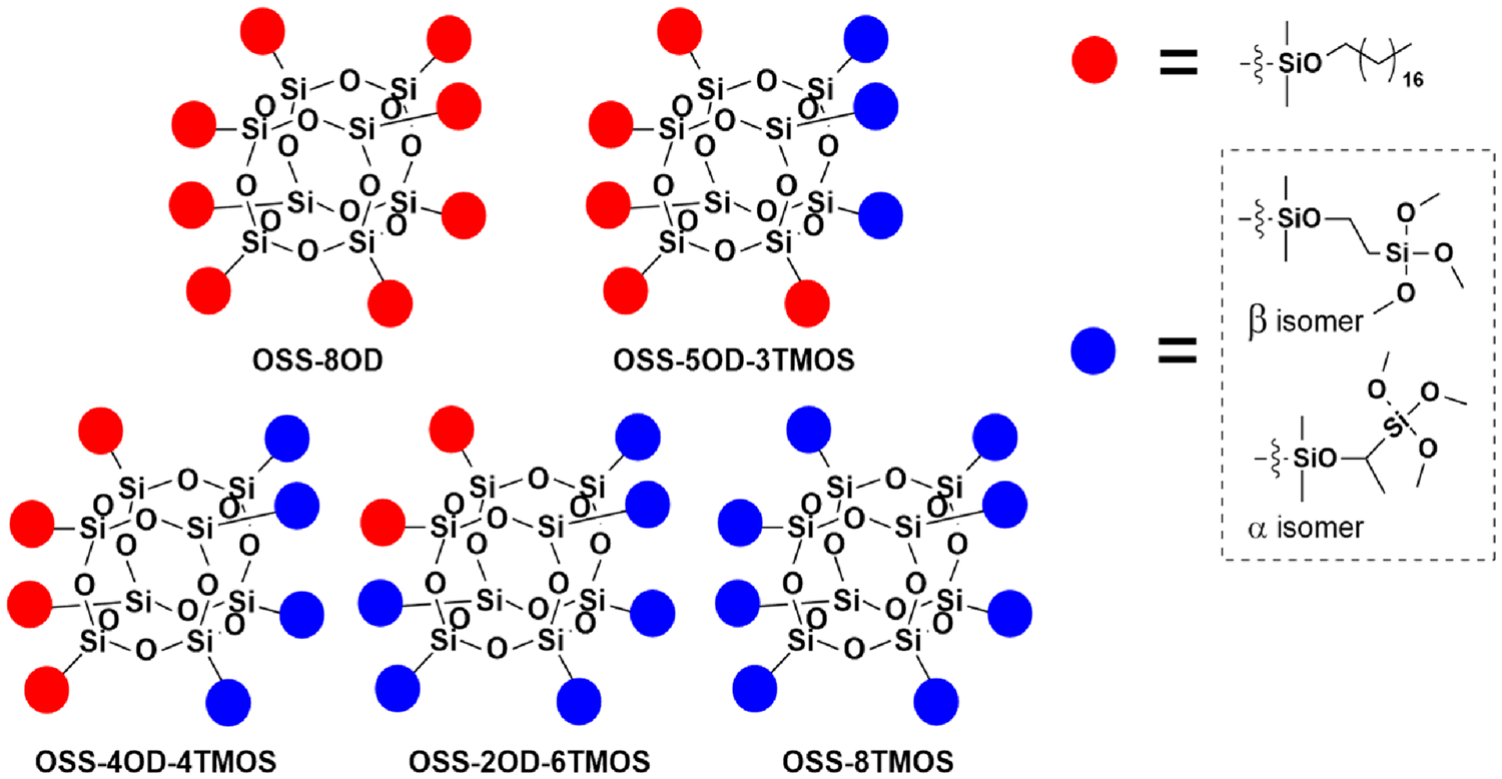
General scheme of the OSS products obtained for this study.

**Figure 4:**
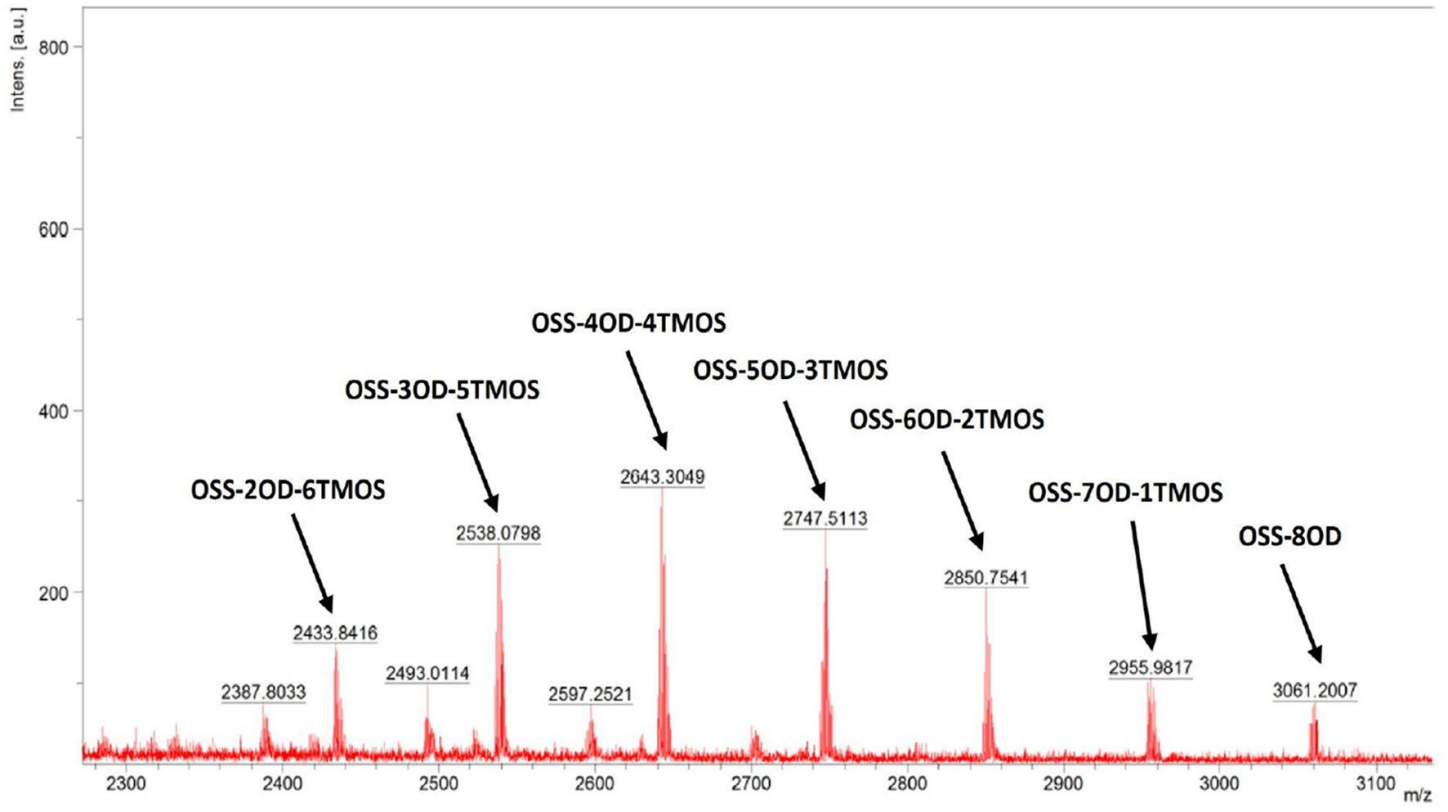
MALDI-TOF-MS analysis of the post-reaction mixture from OSS-5OD-3TMOS synthesis.

The products obtained for the study, prepared in accordance with the procedure from Section 2.3, are presented in Fig. 3. During the synthesis, the reaction was monitored with FT-IR, as the product formation resulted in the disappearance of the characteristic signals at 2141 cm^-1^ and 889 cm^-1^, associated with stretching and bending of the Si–H bond, respectively. The hydrosilylation proceeded with the completion of the reaction (~ 99%). The structure and purity of the compounds were confirmed by ^1^H, ^13^C, and ^29^Si NMR. Hydrosilylation of VTMOS resulted in the formation of 15–20% of α isomer of the corresponding (trimethoxysilyl)ethyl group formed in all the examples, while OD hydrosilylation gave exclusively *β* isomer of the corresponding octadecyl group attached to the spherosilicate siloxy moiety. The MALDI-TOF mass spectrometry analysis confirms the statistical distribution of hydrosilylation products formed. For example, for OSS-5OD-3TMOS, a series of congeners were observed, from a product containing 7 (trimethoxysilyl)ethyl groups and 1 octadecyl group to a product containing 8 octadecyl groups (Fig. 4). This effect of statistical product congeners formation was observed and discussed in more detail earlier^39^.

### Microscopy and spectroscopy

#### SEM–EDS

**Figure 5:**
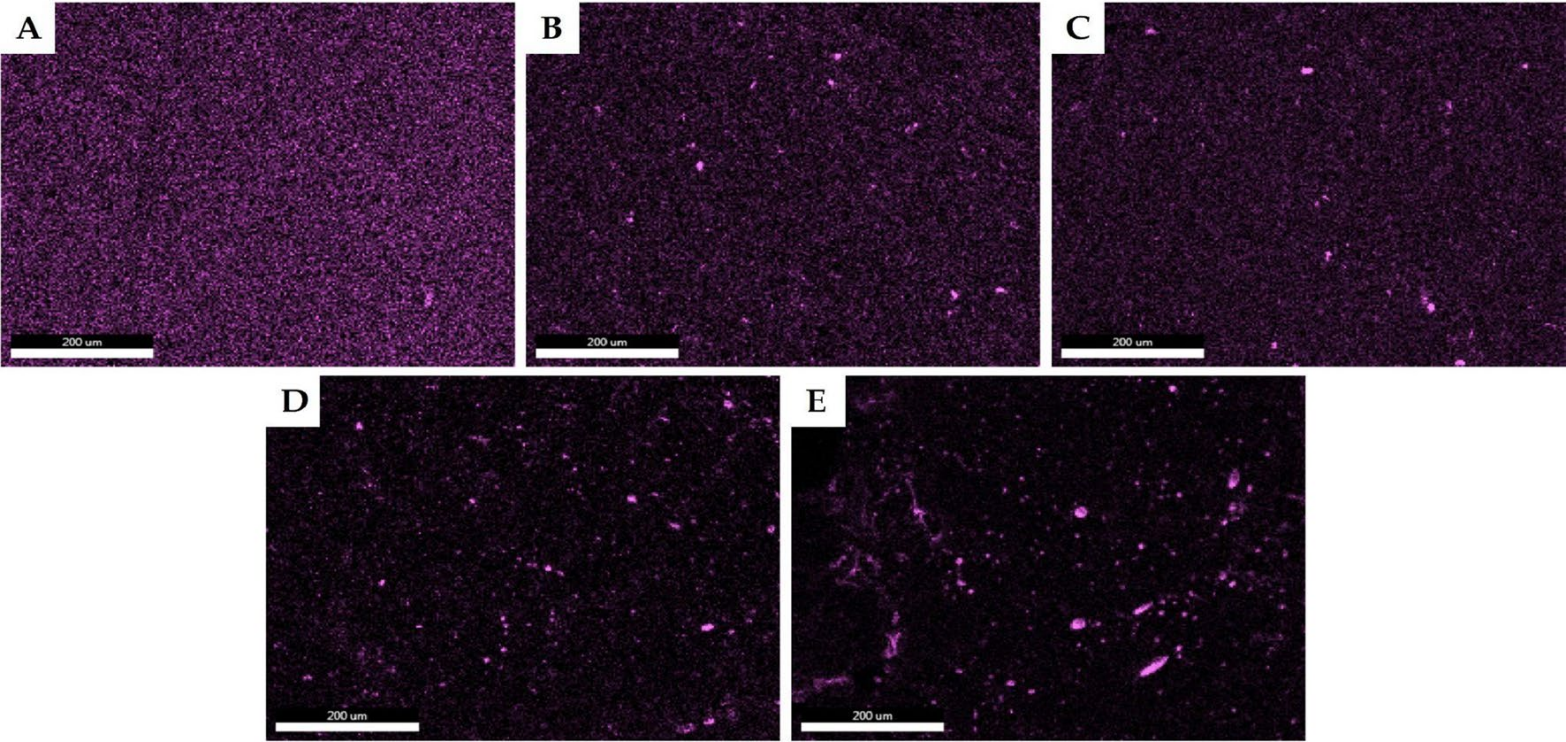
Silicon EDS maps of PLA compositions with 2.5 wt% loading of the studied additives. (**A**) OSS-8TMOS; (B) OSS-2OD-6TMOS; (**C**) OSS-4OD-4TMOS; (**D**) OSS-5OD-3TMOS; (**E**) OSS-8OD.

**Figure 6:**
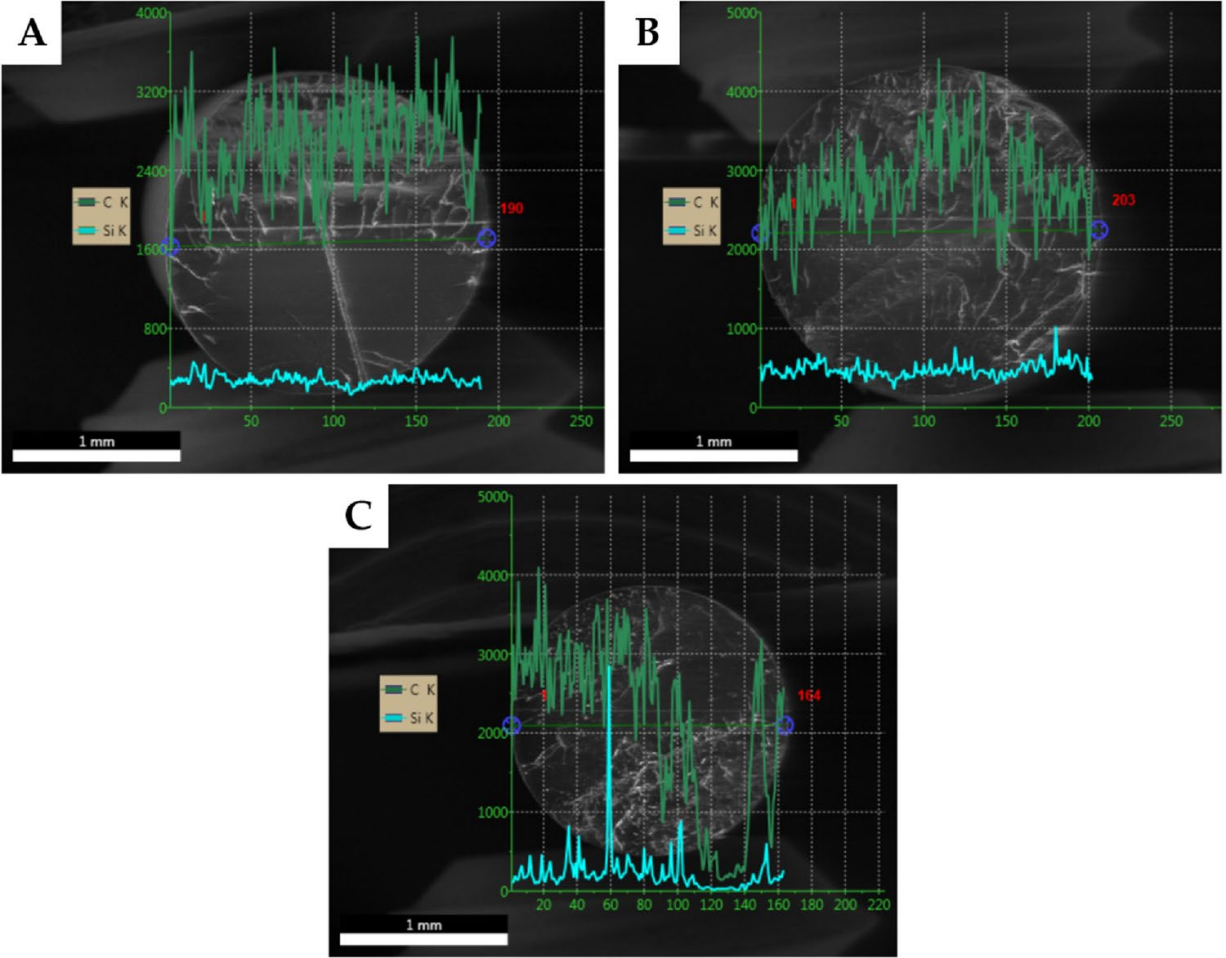
Silicon EDS linear scans of selected fractured filaments fabricated of PLA compositions with 2.5 wt% loading of the studied additives. (**A**) OSS-8TMOS; (**B**) OSS-4OD-4TMOS; (**C**) OSS-8OD.

The miscibility of the additives with the PLA matrix was studied by SEM–EDS microscopic spectroscopy technique. The method has been successfully used previously to assess the compatibility of silsesquioxane and spherosilicate compounds with low-density polyethylene (LDPE)^38^, isotactic polypropylene (iPP)^40^, and polylactide (PLA) matrices^35^, allowing to study structure-, state of matter-, and processing methodology-related aspects of cage siloxane-polymer systems miscibility. The EDS images were taken from a cross-section of the extruded granulate. It can be seen that, besides OSS-8TMOS (Fig. 5A), all the alkoxy silyl-substituted additive systems form larger aggregates (“chicken broth effect”), accompanied by a continuous phase of polymer matrix saturated with the well-dispersed additive (Fig. 5B–D). For OSS-8OD (Fig. 5E), dispersion is minute, and virtually all the additive is aggregated with no micron-sized dispersion presence, showing the least miscibility with PLA. Additionally, EDS scanning was performed in a linear scan mode on a larger scale, that is, fractured samples of the selected fabricated filaments made of PLA compositions with the studied additives (Fig. 6).

The linear scans confirmed that OSS-8TMOS shows the best additive distribution, while PLA composition with OSS-8OD was characterized by significant additive concentration inconsistencies. This effect was present to a much smaller extent for OSS-4OD-4TMOS due to the presence of both well-dispersed and aggregated phases. It is important to note that as for OSS-8TMOS, the additive distribution was seemingly very uniform, however, as further explained for DSC analysis, it does not translate to full miscibility of PLA and the compound itself (see paragraph *Thermal analysis*). Under the polymer melt conditions, many additives for plastics show limited miscibility with the polymer phase in the form of migration capabilities^41^. Based on the physical appearance of OSS-8TMOS, the cause may be its low viscosity and fairly low molecular weight, the latter rising significantly with the number of octadecyl groups in the product molecule.

### Optical microscopy

**Figure 7:**
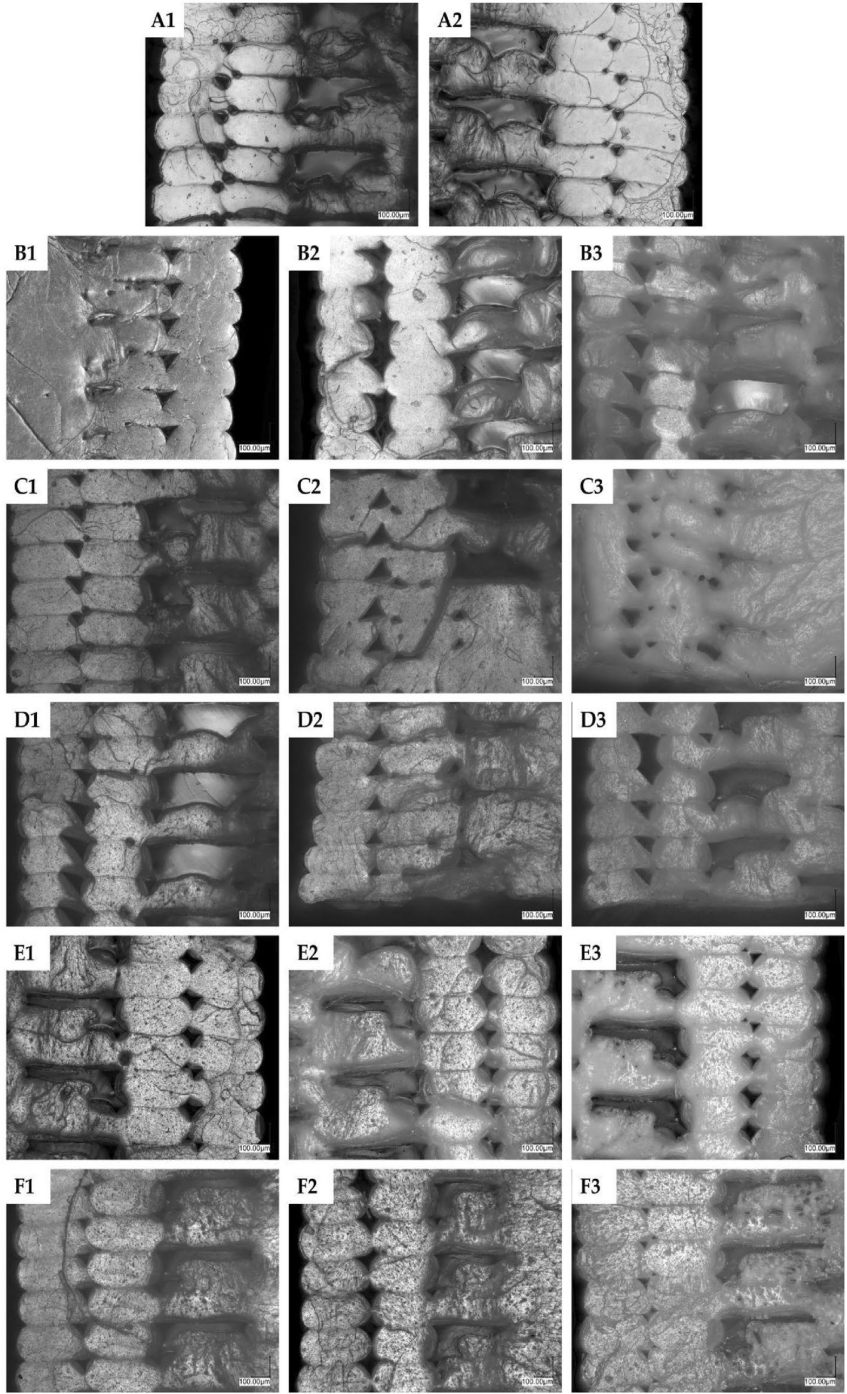
Optical microscopy images of tensile specimens’ cross section after fracture. (**A1**, **A2**) neat PLA, (**B**) OSS-8TMOS, (**C**) OSS-2OD-6TMOS, (**D**) OSS-4OD-4TMOS, (**E**) OSS-5OD-3TMOS, (**F**) OSS-8OD. For samples (**B**–**F**), 1 represents 0.5% additive loading, 2–1%, and 3–2.5%.

Optical microscopy was performed for the 3D printed samples both before and after mechanical analysis (see paragraph *Mechanical analysis*). The analysis of the sample cross-sections after mechanical fracture was an essential tool to understand the impact of the additives on PLA compositions behavior during 3D printing such as interlayer fusion or voids formation, and their modes of mechanical failure (brittle fracture, plastic fracture, debonding) that translate into changes in mechanical strength of the samples under load (Fig. 7). It became obvious that each of the additives affected 3D printing in a slightly different manner, and the loading thereof played a significant role as well. Neat PLA (Fig. 7A1-A2) shows moderate fusion between the layers, as visible by interfacial lines. On top of that, fractures and interlayer voids created by the fracture are present. Upon addition of OSS-8TMOS at low loading (0.5%, Fig. 7B1), a significant improvement of fusion between the layers has been observed, while further addition thereof caused material plasticization (plastic instead of brittle fracture), but it was accompanied by voids formation and cracking of the material. The addition of OSS-2OD-6TMOS at low concentration did not affect fusion by much but caused some plasticization, the effect of which became more visible with increasing loading. At 1%, the fusion improved and at 2.5%, the material showed plastic deformation (plastic draw) upon tensile fracture (Figs. 7C3, 8). OSS-4OD-4TMOS caused cracking and layer debonding within the material, similar to neat PLA, however, some plasticization was observed within the fractured material, especially at the highest loading. Similar conclusions were drawn for OSS-5OD-3TMOS, but at 2.5% loading, the plasticization was significant and no brittle fractures were visible. For OSS-8OD, strong phase separation and porosity introduced into the material by the immiscible additive were observed, together with delamination, but plasticization was visible as well. This factor, together with improved interlayer fusion, seems to be crucial in understanding the mechanical properties improvement of the obtained materials, which is in line with our previous findings on spherosilicate-doped PLA for 3D printing^35^.

**Figure 8:**
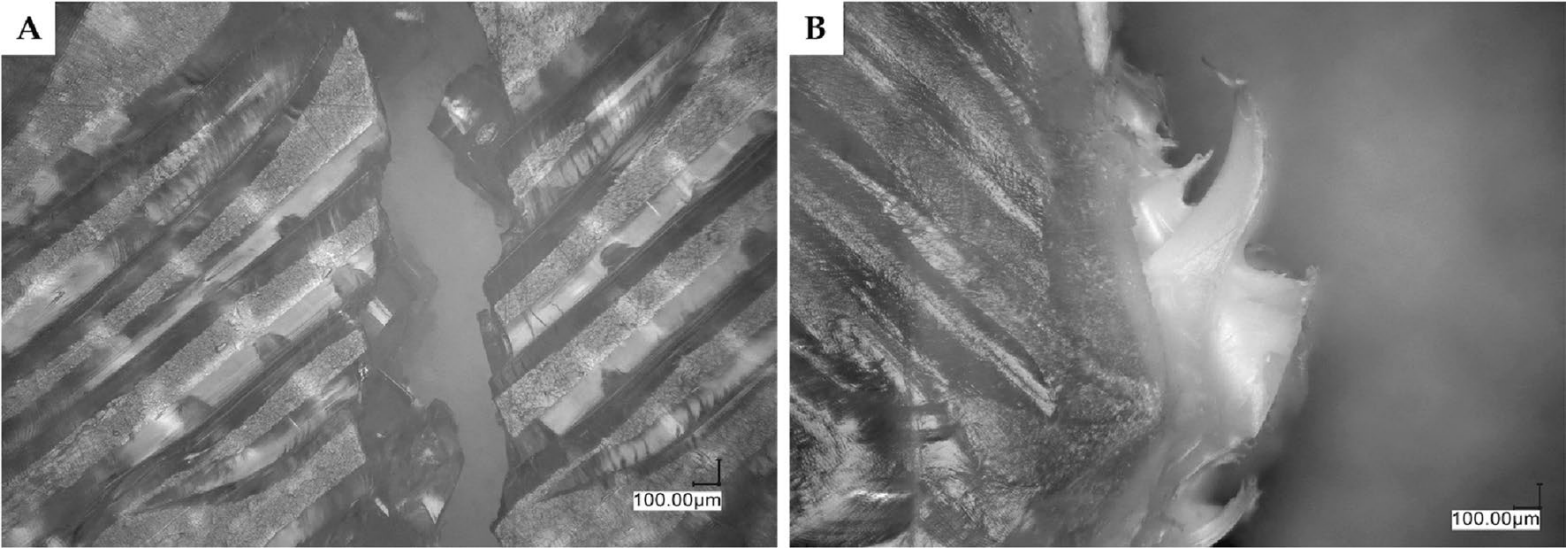
Optical microscopy images of neat PLA (**A**) and PLA with 2.5% OSS-2OD-6TMOS (**B**) after a tensile fracture.

### Contact angle measurements

Table 2 summarises the contact angle measurements, with unmodified PLA showing a value of 70.2°. The modification of the polymer matrix with spherosilicate derivatives containing alkyl (OD) and (trimethoxysilyl)ethyl (TMOS) groups resulted in an increase in contact angle value each time. As expected, allyl groups in the modifier structure contributed to an upward trend due to their non-polar nature and lower affinity to water molecules. This was particularly evident for OSS-8OD, which, due to poor miscibility with PLA, increased the WCA to 82.5° at 0.25% concentration, and then, with increasing loads, the further increase in WCA was much smaller due to the saturation effect, in which the excess additive migrated towards the surface of the sample. OSS-8OD is a hard wax that can solidify on the PLA surface, forming a coating, especially considering its poor miscibility with the bulk polymer. The system with the lowest contact angle values was OSS-8TMOS, which has polar groups and is easily hydrolyzed, resulting in only a slight increase in value, on top of being highly miscible with PLA and residing mostly in the bulk polymer. With increasing modifier concentration, there was a trend observed in all systems where the contact angle value increased.

**Table 2:** Water contact angle [°].

Concentration	Contact angle [°]					
	0.1%	0.25%	0.5%	1%	1.5%	2.5%
PLA neat	70.2 ± 3					
OSS-8TMOS	73.6 ± 1	73.5 ± 1	72.9 ± 1	75.1 ± 1	77.4 ± 1	79.2 ± 1
OSS-2OD-6TMOS	71.3 ± 2	72.7 ± 2	72.8 ± 3	72.5 ± 3	74.6 ± 2	81.7 ± 2
OSS-4OD-4TMOS	76.0 ± 4	72.0 ± 3	74.2 ± 3	79.6 ± 1	78.9 ± 1	82.4 ± 1
OSS-5OD-3TMOS	71.6 ± 2	76.7 ± 2	79.7 ± 2	80.7 ± 4	79.8 ± 3	83.1 ± 3
OSS-8OD	72.2 ± 1	82.5 ± 1	84.6 ± 1	86.2 ± 2	88.4 ± 1	88.6 ± 2

### Thermal analysis

**Figure 9:**
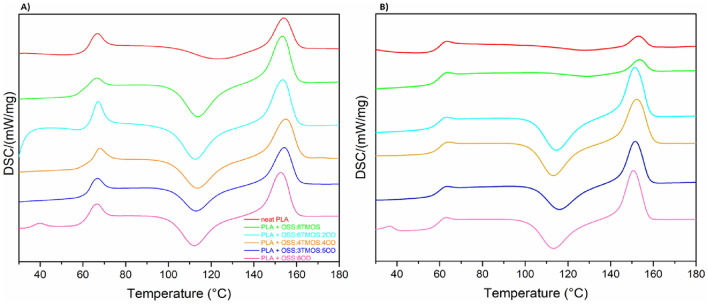
DSC curves recorded for samples of 5% OSS/PLA systems, (**A**) first heating cycle, (**B**) second.

The DSC thermograms of PLA and its blends with the spherosilicate additives revealed that in all the cases, the modified material showed a much more profound cold crystallization event (Fig. 9, Table 3), which may be due to more polymer freedom caused by the plasticizing effect of the additives, as discussed previously in Section “Optical microscopy”, and further in Sections “Rheology” and “Mechanical analysis”. As a result of cold crystallization, the melting event is also expressed by a larger peak area, therefore showing higher sample crystallinity. Moreover, for OSS-8OD, the additive melting event is visible as the additional endotherm at 40℃. For OSS-8TMOS, the cold crystallization effect disappears during the second heating cycle, which is due to phase separation between the additive and PLA, caused by a high migration rate of the low molecular weight, and low viscosity modifier, proving the incompatibility of the two. No significant changes in terms of glass transition or melting temperature have been observed within the studied systems in comparison with the neat polymer, proving that the interactions between the additives and PLA are rather weak and the systems obtained can be considered physical blends of the components.

**Table 3:** DSC data from the first heating cycle, PLA compositions at 5% additive loading.

	Glass transition temperature, T_g_ [°C]	Cold crystallization temperature, T_cc_ [°C]	Melting temperature, T_m_ [°C]
PLA neat	66.5	123.0	154.3
OSS-8TMOS	66.0	113.7	153.7
OSS-2OD-6TMOS	67.2	112.6	153.7
OSS-4OD-4TMOS	67.7	113.4	155.2
OSS-5OD-3TMOS	66.3	113.0	154.6
OSS-8OD	66.9	112.2	152.6

**Figure 10:**
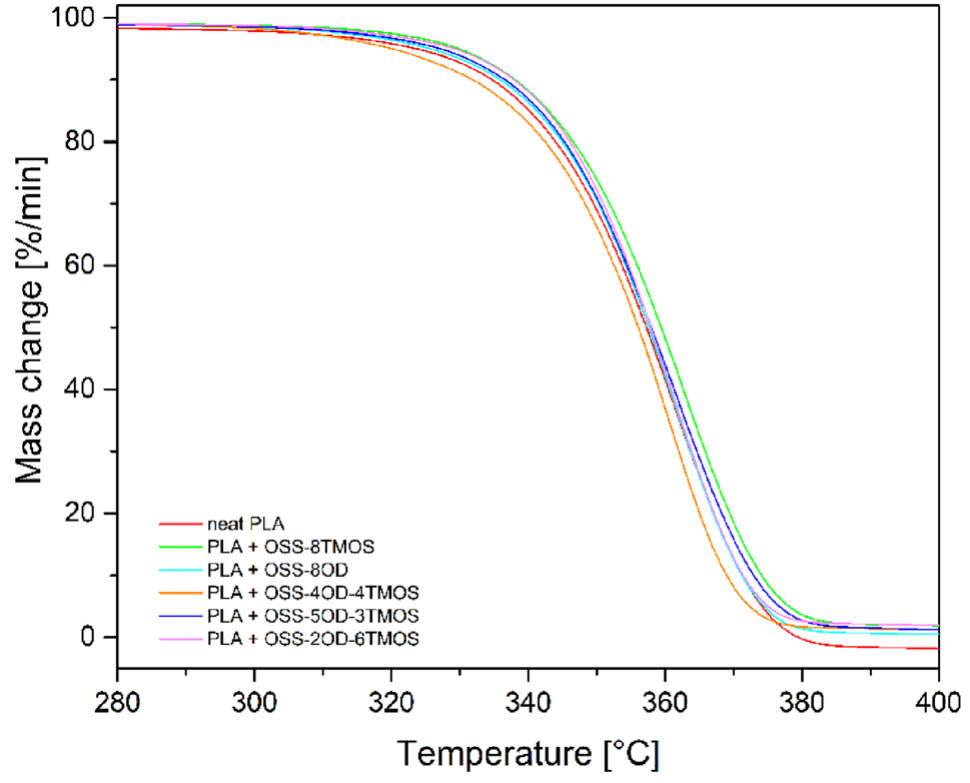
TGA curves, a nitrogen atmosphere, PLA compositions at 5% additive loading.

Thermogravimetry is allowed to confirm the statement mentioned above, as the thermograms of the modified PLA compositions were virtually identical to the ones of neat PLA (Fig. 10). At 5% loading, the additives provided a small (< 10 °C) increase of T_5%_ and T_onset_ parameters, and the T_max_ was unaffected, proving that the additives do not take a part in the mechanism of PLA thermal degradation (Table 4).

**Table 4:** Results of thermogravimetric analysis.

Sample name	5% mass loss temperature, T_5%_ [°C]	Onset temperature, T_onset_ [°C]	Temperature of maximum mass loss rate, T_max_ [°C]
PLA neat	323.7	331.9	362.5
OSS-8TMOS	329.8	337.2	363.2
OSS-2OD-6TMOS	329.4	338.0	361.8
OSS-4OD-4TMOS	320.1	322.4	361.6
OSS-5OD-3TMOS	326.9	333.6	362.1
OSS-8OD	326.1	334.6	361.5

## Rheology

**Figure 11:**
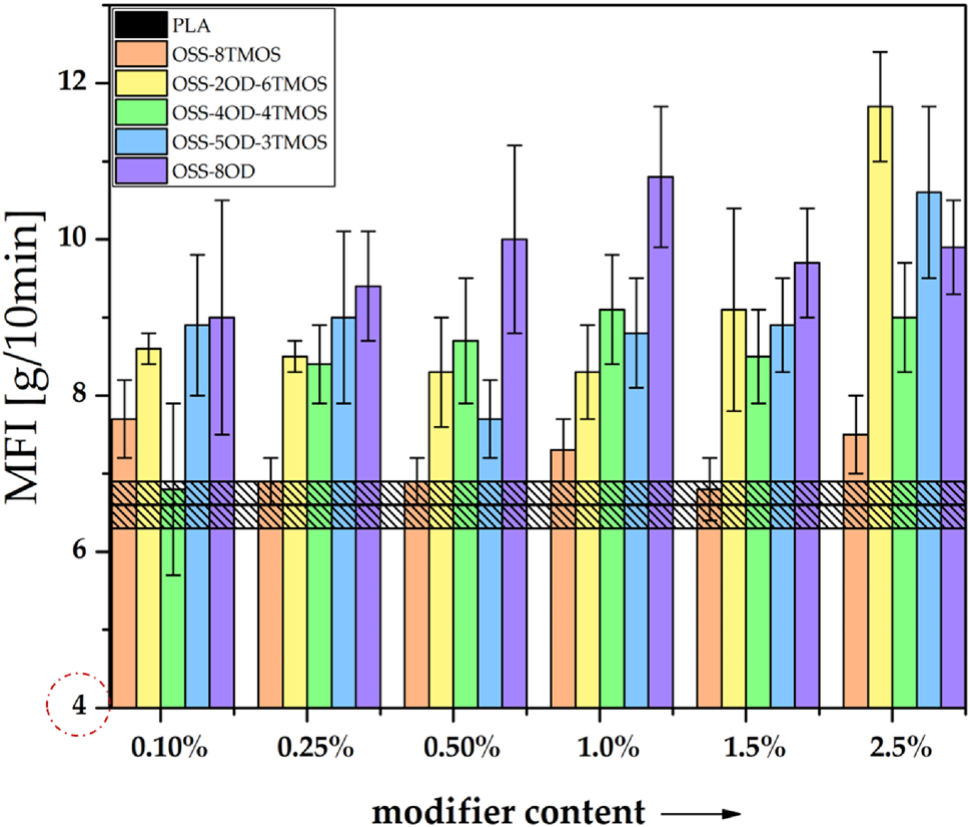
Melt flow index of the obtained PLA compositions doped with OSS compounds.

Melt flow ratio (MFR) was used as a simple tool for the assessment of polymer melt flowability under static load (Fig. 11). It is visible that OSS-8TMOS provides very little increase in MFR that is independent of the additive loading and the results being partially in the range of statistical insignificance. On the other hand, all the alkylated compounds did provide a significant increase of MFR upon addition to PLA. On this basis, the additives take the role of rheology modifier by behaving similarly to paraffin waxes or other synthetic waxes, where the structure of the particular wax plays a role in its miscibility with the polymer phase in the molten state and its impact on the melting behavior of the such obtained system^42^. Therefore, as mostly visible at 2.5% loading, the mixed structure of the additives (both alkoxysilyl and alkylated substituents) provides them lubricating action for the polymer melt under load, as well as miscibility thereof in the polymer melt, and therefore thinning and plasticizing properties.

## Mechanical properties

### Tensile tests

**Figure 12:**
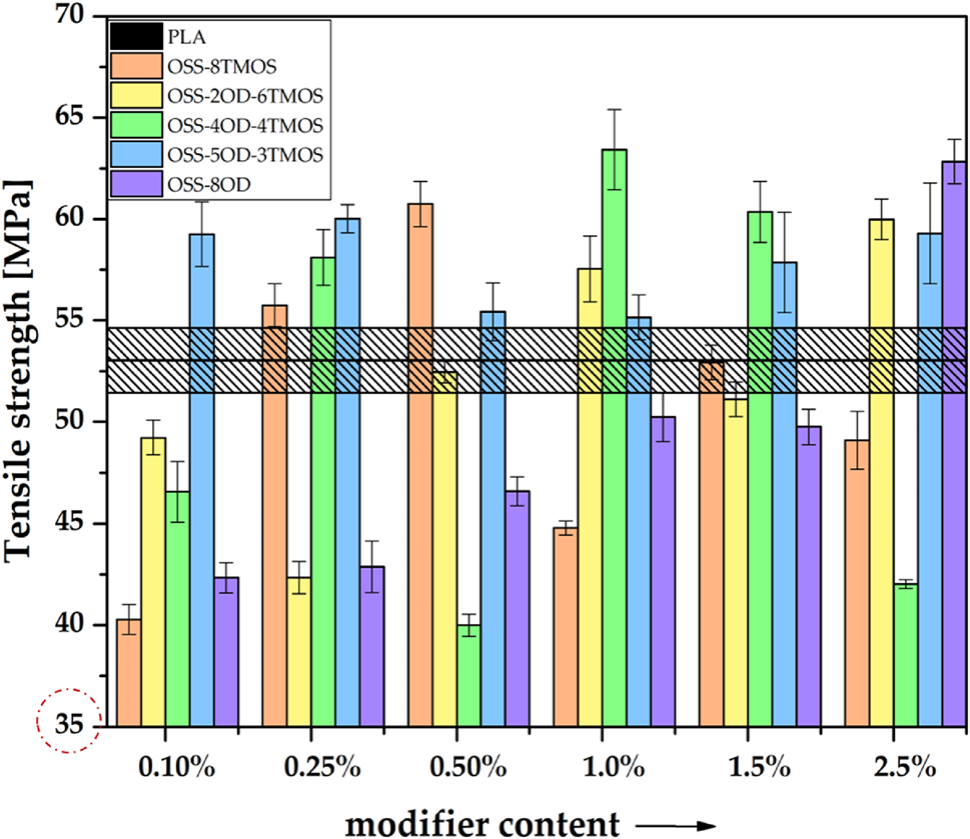
Tensile strength of 3D printed PLA/OSS specimens.

**Figure 13:**
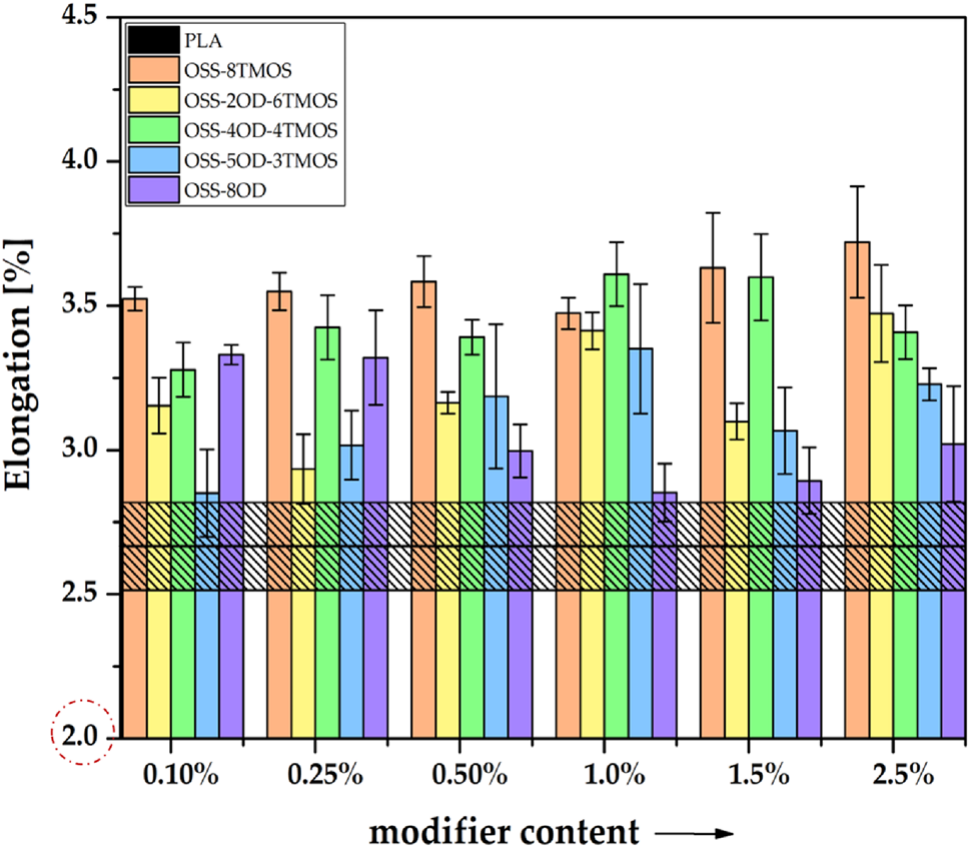
Elongation at maximum load of 3D printed PLA/OSS specimens.

**Figure 14:**
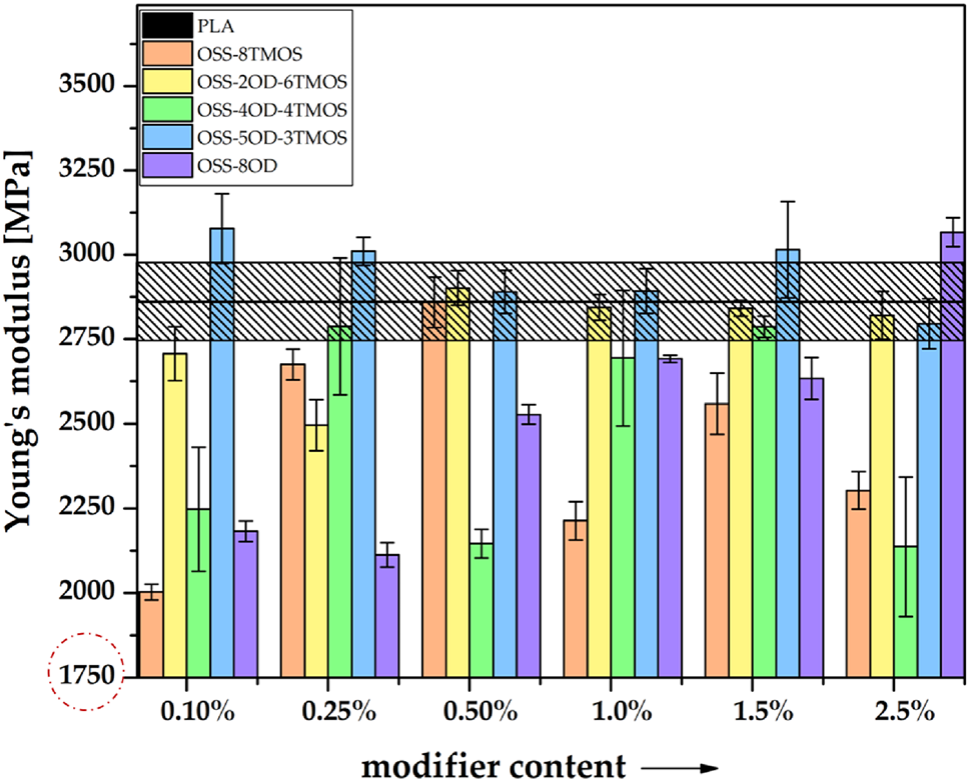
Young’s modulus of 3D printed PLA/OSS specimens.

Tensile tests were performed to investigate the effects of the additives on the mechanical properties of the obtained materials. A strong impact can be seen both derivative- and loading-wise, but besides OSS-8OD, there was no simple correlation between the additive loading and mechanical properties of the such obtained PLA compositions, as the microscopy analysis (see *Optical microscopy*) revealed that the OSS compounds affect several aspects of the material microstructure and mechanical behavior under load, e.g. interlayer fusion, delamination, cracking, phase separation, and polymer phase plasticization (Figs. 12, 13, 14). The observed effects are therefore non-linear and more difficult to discuss than those of rigid material such as injection moulded specimens, however, a general conclusion can be drawn that the tensile strength of the specimens is primarily a result of the print quality (rigidity of the internal structure), and possibly to some degree, of PLA plasticization. For OSS-8TMOS, the best results were obtained at 0.5% loading, where, as proven by microscopy, improved interlayer fusion was achieved. Additionally, at all loadings, significant improvement in plastic elongation was observed (Fig. 13). For OSS-2OD-6TMOS, good plasticization (confirmed by increased elongation and reduced Young’s modulus, Figs. 13, 14) together with improved fusion were achieved at high loadings, which translated to increased tensile strength most notably at 2.5%. OSS-4OD-4TMOS and OSS-5OD-3TMOS caused debonding and cracking of the material, alongside plasticization that was becoming more visible with increasing loading, on top of phase separation becoming visible for the latter additive. Therefore, speaking in general for most of the additives studied, positive effects (plasticization, fusion) are accompanied by negative ones (cracking, debonding) and therefore there is a transition from one mode of failure into another, depending on the amount of a given OSS compound used. For OSS-8OD, poor miscibility caused phase separation of the polymer, which introduced porosity visible by microscopy. However, at 2.5%, brittle cracking was eliminated entirely and voids share was minimised, resulting in notable increase of tensile strength, while Young’s modulus being similar to that of neat PLA suggests that, instead of plasticization, the phase separation phenomenon was rather the factor playing the more significant role in improving the mechanical behaviour of the material under load. This finding confirms the physical nature of the blend speculated above.

### Impact strength

**Figure 15:**
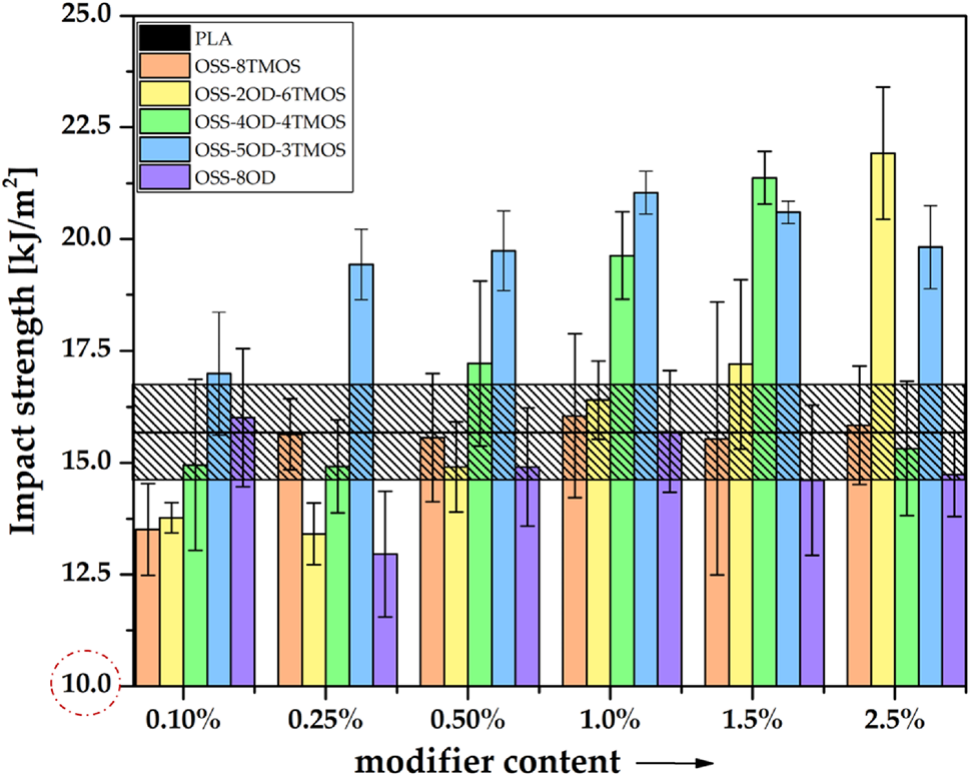
Impact strength of POSS/PLA in 3D printing.

In the case of the impact tests, similar to the mechanical test results presented earlier, a strong influence of both the type of spherosilicate used and its concentration on the ability to carry mechanical loads is evident. The results obtained are presented in Fig. 15. Plasticization causes a decrease in the brittle fracture of the samples, thus allowing for more energy to be absorbed by the sample during impact (Table 5). A particular increase in ductility already at low concentrations occurs for PLA/OSS-5OD-3TMOS system, which is probably due to both plasticization and phase separation between the additive and the polymer phase, as seen by optical microscopy, as a result of the limited miscibility of the phases^43^ (see Section “Optical microscopy”). As the mobility of PLA macromolecules was enhanced by the plasticizer, resulting in a reduction of the effects of brittle fracture, more energy could be absorbed. Depending on the miscibility of different OSS compounds studied in this work, the effect was observed at higher loadings as less alkylated, more miscible additives were tested (OSS-4OD-4TMOS, OSS-2OD-6TMOS). The impact strength values determined for other systems oscillate within the uncertainty range determined for the reference sample.

**Table 5:** Average energy absorbed by the sample upon impact for PLA and its composite systems.

Concentration	Energy absorbed during impact [%]					
	0.1%	0.25%	0.5%	1%	1.5%	2.5%
PLA neat	2.51 ± 0.17					
OSS-8TMOS	2.16 ± 0.2	2.50 ± 0.1	2.49 ± 0.2	2.65 ± 0.3	2.49 ± 0.5	2.54 ± 0.2
OSS-2OD-6TMOS	2.27 ± 0.2	2.15 ± 0.1	2.39 ± 0.2	2.63 ± 0.1	2.75 ± 0.3	3.51 ± 0.2
OSS-4OD-4TMOS	2.37 ± 0.1	2.39 ± 0.2	2.76 ± 0.3	3.14 ± 0.2	3.42 ± 0.1	2.31 ± 0.4
OSS-5OD-3TMOS	2.88 ± 0.1	3.31 ± 0.1	3.17 ± 0.2	3.44 ± 0.1	3.32 ± 0.1	3.16 ± 0.1
OSS-8OD	2.56 ± 0.3	1.88 ± 0.4	2.39 ± 0.2	2.51 ± 0.2	2.27 ± 0.3	2.36 ± 0.2

## Conclusions

Additive technologies (3D printing) require the development of materials dedicated strictly to the processing properties of 3D printers. For this reason, each time when designing a material, it is necessary to characterize the mechanical and thermal properties as comprehensively as possible or to assess the quality of the printout. The introduction of silsesquioxanes with variable functional group stoichiometry, presented in this study, is a useful tool for precise modeling of the indicated properties. Materials with a concise internal structure of the printout are a product for maintenance systems, production of spare parts, and parts of machines and devices in short-series production. In particular, objects with a solid internal structure are used in printing elements for the transport of gases or liquids (e.g. hydraulic fittings). This work presented the attempts at the application of novel compounds of octaspherosilicate (OSS) type as the additives for controlling the properties of PLA for FDM 3D printing, as a continuation of our previous studies on this matter. The effects of the additive molecular structure and loading were discussed with respect to the additive-polymer compatibility (miscibility), and the thermal, mechanical, and surface properties of such obtained materials, as well as their print quality and rigidity. The alkoxysilyl groups provided good miscibility of the additives with PLA, while on the other hand, long alkyl chains were too apolar for suitable compatibility with the polyester-type polymer. However, the right proportion of the two substituents within an OSS compound allowed for obtaining an additive of superior properties when introduced into PLA. Especially interesting properties were obtained for OSS-2OD-6TMOS, where improved interlayer fusion within the printed material, plasticization of the material, and therefore increased mechanical toughness were achieved, on top of enhanced crystallization ability and hydrophobic properties.

## Data Availability

The datasets used and/or analysed during the current study available from the corresponding author on reason-able request.
